# Single Cell Analysis of Human RAD18-Dependent DNA Post-Replication Repair by Alkaline Bromodeoxyuridine Comet Assay

**DOI:** 10.1371/journal.pone.0070391

**Published:** 2013-08-06

**Authors:** Mónika Mórocz, Himabindu Gali, István Raskó, C. Stephen Downes, Lajos Haracska

**Affiliations:** 1 Institute of Genetics, Biological Research Centre of Hungarian Academy of Sciences, Szeged, Hungary; 2 Biomedical Sciences Research Institute, School of Biomedical Sciences, University of Ulster, Coleraine, Londonderry, Northern Ireland; University of Massachusetts Medical School, United States of America

## Abstract

Damage to DNA can block replication progression resulting in gaps in the newly synthesized DNA. Cells utilize a number of post-replication repair (PRR) mechanisms such as the RAD18 controlled translesion synthesis or template switching to overcome the discontinuities formed opposite the DNA lesions and to complete DNA replication. Gaining more insights into the role of PRR genes promotes better understanding of DNA damage tolerance and of how their malfunction can lead to increased genome instability and cancer. However, a simple and efficient method to characterise gene specific PRR deficiencies at a single cell level has not been developed. Here we describe the so named BrdU comet PRR assay to test the contribution of human RAD18 to PRR at a single cell level, by which we kinetically characterized the consequences of the deletion of human RAD18 on the replication of UV-damaged DNA. Moreover, we demonstrate the capability of our method to evaluate PRR at a single cell level in unsynchronized cell population.

## Introduction

Successful progression through S-phase and completion of DNA replication are continuously challenged by various endogenous and exogenous DNA-damaging agents. Stalling of replication at unrepaired DNA lesions can result in discontinuities in the newly synthesized DNA. Cells have evolved mechanisms to overcome these discontinuities, which has been named as post-replication repair referring that it can also operate after the majority of DNA has been replicated and not only in the S-phase but even in the G2 phases, as well [1]–[5]. During PRR, DNA lesions can be bypassed either directly by translesion synthesis polymerases, which can incorporate nucleotides opposite the damaged bases [6], or indirectly by template switching, which facilitates copying from the newly synthesized sister strand [7]–[9]. The current understanding of PRR processes highlights the regulation of these pathways in a RAD6/RAD18-dependent manner [5], [10], [11]. The absence of RAD18 results in defective post-replication repair as revealed by the conventional alkaline sucrose gradient centrifugation based PRR assay and hypersensitivity to multiple mutagens such as UV irradiation, cross-linking agents or alkylating agents, which was demonstrated in yeast, chicken, mouse and human cells [12]–[16]. Homologous recombination dependent pathways can also provide an alternative means for PRR [12], [17]–[19]. However, the exact molecular mechanisms of PRR and its genetic requirements have not been fully understood partly because of the lack of simple and highly sensitive PRR methods.

The comet assay or single-cell gel electrophoresis is a rapid, sensitive method that measures discontinuities in the genomes of individual cells [20], [21]. The basic comet assay and its variants can measure the amount of single- and double-strand DNA breaks, apurinic-, apyrimidinic-, and alkali-labile sites, DNA cross-links, base damages and apoptotic nuclei [22]–[28]. In the basic alkaline comet assay method, a low amount of cells is embedded in agarose on a microscope slide, and lysed to remove proteins, membranes and cell constituents. The DNA is left to unwind in alkaline solutions and electrophoresed in the same buffer. During the short electrophoresis, the high molecular weight DNA is unable to move in the agarose matrix and forms the round head of a comet-like nucleoid (as the nucleus is referred to after lysis), containing naked DNA. As a consequence of alkaline conditions, the unwound loops and the fragmented, low molecular weight DNA are pulled out by the electric current and is forced to migrate towards the anode forming a tail-like structure, resulting in a comet-like form [29]. After neutralisation and staining with fluorescent dye the comets are visualised by fluorescent microscopy. The amount of discontinuous DNA represented by comet tail DNA can be quantitatively measured by visual scoring or software-guided analysis. The basic comet assay has also been extended to various applications such as to visualize defined regions in the genome by combination with fluorescence in situ hibridization or pulse labelling replicating DNA with a thymidine analogue bromodeoxyuridine (BrdU).

BrdU is frequently used DNA replication precursor analogue to mark DNA during DNA synthesis and thus has a long history of use in studies of DNA replication and repair. It is used to density label DNA, monitor ratio of S-phase cells in asynchronous cell population during cell cycle progression in flow cytometry, visualize newly synthesized DNA by immunohistochemistry, and measure the speed of replication in DNA fiber assay [30]–[33]. Owing to its bromine side group, BrdU can be potentially harmful to cells leading to DNA alteration in certain experimental conditions, but this effect was detected at high ratio of substitution of thimidine to BrdU and when combined with altered DNA conformation or secondary stressors such as high dose of IR- and UV-irradiation [34], [35]. If applied for short pulse times, however, BrdU had no significant effect on the length of S-phase and the rate of replication fork progression. In contrast to the novel thimidine analogue EDU, which induced a profound DNA damage response, BrdU has not affected the cell cycle distribution of human fibroblast cells and has not altered significantly the level of DNA damage response markers such as formation of γH2AX, RPA32, and 53BP1 foci in mouse embryonic stem cells [31], [36].

Previously, a comet assay variant to detect DNA maturation by pulse labelling of the replicating DNA with a thymidine analogue bromodeoxyuridine (BrdU) was developed, and it was applied to identify replication defects of premalignant cells [37], [38]. It was shown that the BrdU comet assay can be used to detect defective DNA maturation in DM87 and caffeine-sensitive PRR in SVM84 indian muntjac cell lines upon UV irradiation confirming the findings having been previously demonstrated by other established techniques.

To extend this study and to provide a single cell based sensitive PRR method here we describe a so named BrdU comet PRR assay, by which we characterised human RAD18 in PRR at a single cell level. By comparing the efficiency of PRR in RAD18^−/−^ and RAD18^+/+^ human HCT116 cells without and upon UV damage we revealed a severe delay in DNA elongation and the completion of DNA replication after UV irradiation in RAD18 deficient cells. These data extended the previous observations for the role of RAD18 in DNA damage tolerance as well as evaluated the BrdU comet PRR assay.

## Materials and Methods

### Cell Lines

HeLa cells were cultured in Dulbecco’s modified Eagle medium (DMEM) supplemented with 10% fetal bovine serum, and cultures were incubated at 37°C in humidified chambers with 5% CO_2_. Three isogenic HCT116 cell lines [39] were used: 1, HCT116(Wt), 2, HCT116(RAD18^−/−^), and 3, HCT116(RAD18^−/−/^+RAD18), which is a HCT116(Rad18^−/−^) cell line expressing RAD18 for complementation and it was formerly referred as HCT116(clone28). The growth conditions were the same as previously described.

### BrdU Labelling, UV Exposure and Chase

Exponentially growing cells were plated at a density of 2×10^4^ cells/well of a six-well plate in complete DMEM medium and grown for 24 h. Growth media were replaced with fresh medium containing 20 µM BrdU at 37°C for 20 min. Cells were next washed two times with PBS, mock treated or exposed to 20 J/m^2^ UV-C light (or at indicated doses in dose-effect experiments) and subsequently chased in fresh medium containing 200 µM of each of the four dNTPs until the indicated time points.

### BrdU Comet Assay

#### Slide preparation

For proper attachment of the agarose layer to microscope slides (fully frosted, Menzel GMBH, Germany) a precoating was performed. The day before the experiments the frosted microscope slides were individually dipped up to the frosted end into hot 1% agarose (Sigma-Aldrich), dissolved in water. After draining the excess agarose, the backsides of the slides were wiped and the slides were left to air dry horizontally in a dry place. After drying the agarose gives an almost invisible crystal-clear coating on the surface that serves as a good anchoring for the next agarose layers. Slides can be stored for weeks in a dry place; alternatively, sandblasted, fully frosted microscope slides can be used.

#### Embedding of cells

Hundred and sixty microliters of 1% normal agarose (dissolved in water, Sigma-Aldrich, Germany A9539) were spread on each precoated slide as a basal agarose layer just before collecting the cells. The slides were covered with a 24×32 mm coverslip (Menzel GMBH, Germany), and kept for 3 minutes at 4°C to solidify. The medium was removed after chase and cells were washed with PBS, trypsinized and collected in 1 ml ice-cold PBS. The cells were pelleted by centrifugation and resuspended in 70 µl of 0.75% low-melting agarose (Sigma-Aldrich, A9414) dissolved in PBS kept at 37°C. The coverslip was removed from the slides and the cell suspension in agarose was spread onto the first agarose layer, the coverslip was replaced on top and the slide was left at 4°C for 3 min to solidify. After removal of the coverslip, slides were placed in ice-cold lysis solution (2.5 M NaCl, 100 mM EDTA, 10 mM Tris [pH 10], 1% Triton X-100 and 0.5% N-lauroylsarcosine sodium salt (Sigma-Aldrich), with the detergents added freshly before use) for 90 min in a Coplin jar to remove membranes and proteins. After lysis the slides were placed horizontally side by side in a glass tray and washed for 3×5 min by gently layering with PBS and drained by careful tilting on a paper towel. This step removes any remaining cell debris, results in clean background and reduces aspecific antibody adhesion during immunostaining.

#### Electrophoresis

Slides were placed side by side into a horizontal electrophoresis tank containing ice-cold alkaline electrophoresis buffer (0.3 M NaOH, 1 mM EDTA, pH 13), the empty places filled with empty slides. The DNA was left to unwind for 40 minutes in this ice-cooled electrophoresis buffer. The electrophoresis was subsequently conducted at 1 V/cm (25 V, 300 mA) for 20 minutes in the same buffer at 8–10°C. Following the electrophoresis the slides were placed horizontally side by side on a glass tray, and washed for 3×5 min by gently layering with neutralization buffer (0.4 M Tris-HCl, pH 7.4) and drained by careful tilting on a paper towel. Before immunostaining the slides can also be stored overnight after being layered with 50 µl neutralization buffer, covered with coverslips in a humidified box at 4°C.

#### Immunostaining

Slides were washed with two changes of PBS for 5 minutes each and blocked with PBS+BT (PBS containing 0.1% BSA and 0.1% Tween20), for 30 min by horizontal layering of solutions onto the surface of the agarose at room temperature. The slides were incubated with 30 µl/gel of rat monoclonal anti-BrdU (1∶750, Ab-Direct Serotech) in the dark in a humidified box at room temperature for 1 h. The excess of primary antibody was washed off with three changes of PBS and once with PBS+BT, and probed with 30 µl/gel of secondary antibody (Alexa Fluor 488-labelled goat anti-rat antibody (1∶750); Molecular Probes, Inc.) for 1 h and washed off as described above. The slides were counterstained with ethidium bromide, covered with coverslips, placed on a glass plate into a humidified box and stored at 4°C until microscopy.

#### Microscopy

Visualisation of comets was made at a final magnification of 400x using a fluorescence microscope (Zeiss, Axioscope, Germany) equipped with an Axiocam Digital Camera. The Komet 5.0 video image analysis software (Kinetic Imaging Ltd., Liverpool, UK) was used to analyze DNA fragmentation. This software is designed to differentiate comet head (nuclei) from tail (damaged DNA). Free comet analysis software is available for downloading at http://autocomet.com/main_home.php. The images of 50 randomly chosen nuclei per slide were captured and analyzed. The amount of discontinuous DNA migrating in the tail was shown as the percentage of total fluorescence (% DNA in tail) for each nucleus. This value was then averaged over the 50+50 nuclei measured in the duplicate slides, and the mean of minimum 3 independent experiments ± SD was calculated to generate each data point.

## Results

### Alkaline BrdU Comet Method can Function as a Sensitive Post-replication Repair Assay

**Figure 1:**
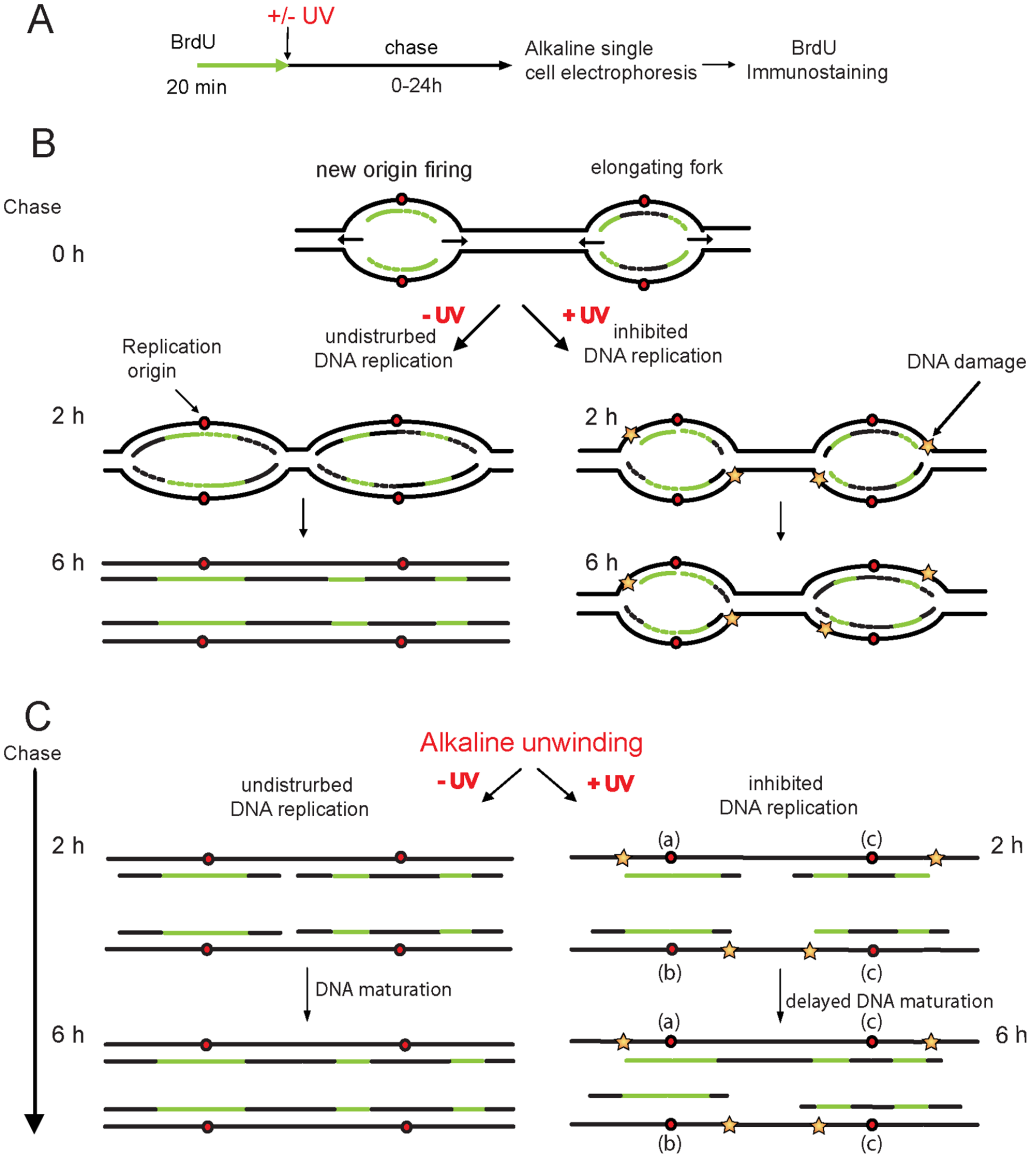
Main principle of the alkaline BrdU comet PRR assay. (**A**) Experimental setup. (**B**) Chematic illustration of neighbouring origins and bidirectional way of replication fork movement. BrdU incorporates into newly replicated DNA strands during short pulse labelling. As replication progresses, the labelled strands move apart from the replication fork during the chase period and are incorporated into high molecular weight DNA. In the untreated cells (-UV) the discontinuities of DNA strands are abolished gradually by ligation of DNA strands elongated from neighbouring origins in a process called DNA maturation. In the presence of DNA adducts caused by UV irradiation (+UV), replication fork movement is inhibited and the nascent DNA remains discontinuous for a longer time. (**C**) At early stages of replication of an undamaged template, the short stretches of newly replicated DNA can be separated from the template strand by alkaline unwinding and visualised as comet tail DNA after short alkaline electrophoresis followed by immunostaining with anti-BrdU antibody. Two different types of events are presented as examples with neighbouring replication origins: labelling of new origin firing and an elongating fork. As the replication fork elongates on undamaged template and DNA maturation is completed by the ligation step, the DNA becomes a continuous, high molecular weight molecule, forming the comet head. On damaged templates DNA replication is inhibited and the DNA remains fragmented, the labelled fragments migrate into the comet tail and can be detected as low molecular weight fragments even at late time points. Examples of detectable labelled fragments are shown as (a) termination of a new origin firing, (b) termination of elongation and (c) inhibition of elongation.

We aimed to develop a PRR method based on the principle that if the newly replicating DNA is pulse labelled for a short period with thymidine analogue BrdU the elongation of the replication forks and discontinuities left in the newly synthesized DNA can be measured at different times using alkaline comet assay followed by BrdU immunostaining (Figure 1A). During an undisturbed replication process, when the elongating DNA ends synthesized from neighbouring replicons abut, DNA ligase seals the gaps of DNA fragments and the nascent DNA becomes continuous [40]. This process is also referred to as DNA maturation of replicative intermediates for both the nascent leading strand DNA and Okazaki fragments [41], [42] (Figure 1B). Before the DNA maturation is completed the nascent DNA fragments can be unwound under alkaline conditions, indicating incomplete replication and the presence of single-stranded gaps in the newly replicated DNA (Figure 1C, left panel). The nascent DNA fragments can be separated from the template strand by alkaline unwinding and electrophoresis and can be visualised as comet tail DNA after immunostaining of the incorporated BrdU. In the presence of a DNA damaging agent, when replication fork movement is inhibited and DNA elongation is blocked, the newly synthesized DNA remains fragmented for a longer time (Figure 1C).

We have successfully used this experimental setup for exploring the effect of various DNA damaging agents on PRR. For UV-light, we took into account that the incorporation of high level of halogenated nucleotide analogue can sensitize against UV irradiation and cause DNA strand breaks. This effect was described at high UV dose after both DNA strands were continuously labelled with halogenated nucleotide analogues present at high ratio during several rounds of DNA replication [35]. Since we used low UV-dose and short pulse labelling at a low ratio of BrdU, we ascertained the absence of these artefacts under our experimental condition by comparing experiments with BrdU pulse labelling before and after-UV treatment, but we did not detect additional single or double strand break formation when BrdU-pulse was applied before UV treatment (data not shown). For further experiments we choose the BrdU labelling before the DNA damage treatment because the labels were more consistent and also allowed to use the same experimental conditions for DNA fiber experiments, by which one can also follow immediate DNA damage bypass in parallel with monitoring PRR [30], [43].

### Alkaline BrdU Comet PRR Assay is able to Differentiate Normal and DNA Damage Disturbed Replication

**Figure 2:**
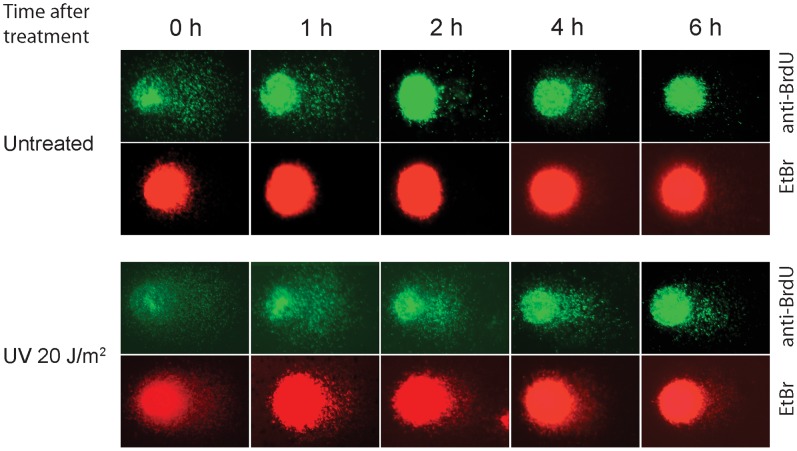
Alkaline BrdU comet PRR assay images of untreated and UV-irradiated HeLa cells representing the progression of replication as detailed in Figure 1. The cells were pulse labelled with BrdU before irradiation with 20 J/m^2^ UV-C or mock treatment. After the indicated time (0–6 h) single-stranded DNA fragments were separated from matured DNA by alkaline single cell electrophoresis followed by immunostaining using fluorescent anti-BrdU antibody (green) or staining with ethidium-bromide (red). The much higher sensitivity of the BrdU comet assay as compared to the basic comet assay was demonstrated by showing the anti-BrdU stained and ethidium bromide counterstained images of the same cells.

Discontinuities in newly synthesized DNA should disappear by the end of DNA replication. However, DNA elongation slows down significantly at the site of lesions and thus the discontinuities exist for a longer period of time in cells with damaged DNA. To test the ability of the BrdU comet method to differentiate replication maturation in damaged and undamaged cells, we cultured exponentially growing HeLa cells and pulse-labelled them in the presence of BrdU for 20 min, washed and UV-irradiated or mock treated. BrdU-labelled DNA was chased in the presence of 4xdNTPs for 0, 2, 4, and 6 h. At indicated time points cells were collected and examined by alkaline BrdU comet assay. The incorporated BrdU was immunostained using a specific anti-BrdU antibody followed by an ethidium bromide counterstain of the slides. We found that assembly of fragments occurred continuously in a time-dependent manner as visualized by a decrease in the amount of comet tail DNA upon BrdU immunostaining (Figure 2). The process of DNA maturation was found to be complete after 6 h in the untreated HeLa cells. During this process the amount of high molecular weight DNA constituting the comet head increased with time and became compact with bright fluorescence, reflecting the completed replication. However, completion of replication was severely inhibited even after 6 h in cells irradiated with UV light. Notably, the ethidium bromide counterstain detected no comet tails in cells with undisturbed DNA replication, and less intense tails in damaged cells as compared to the detection with anti-BrdU antibody indicating the much better applicability of the BrdU comet method in measuring DNA maturation kinetics during replication than the basic comet assay.

### Alkaline BrdU Comet PRR Method Provides Greater Sensitivity than the Standard Alkaline Comet Assay

**Figure 3:**
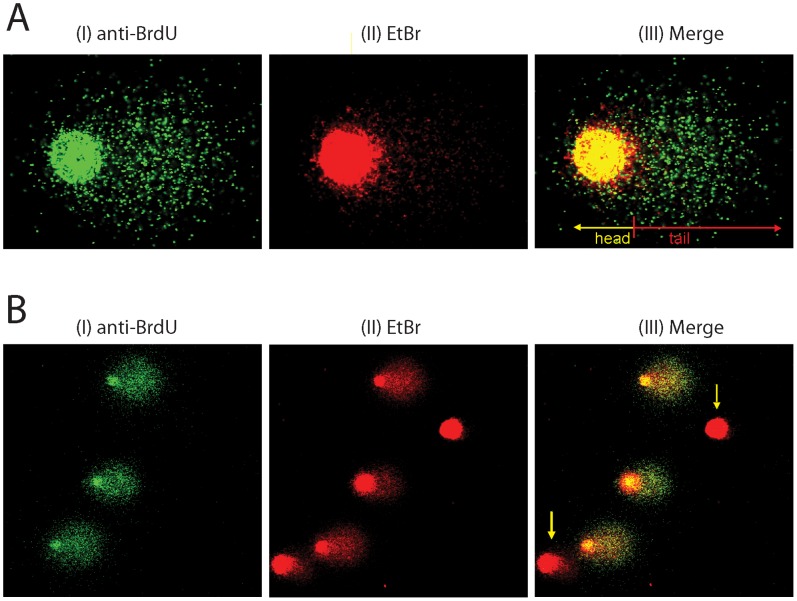
Alkaline BrdU comet PRR images of HCT116(RAD18^−/−^) cells after UV irradiation. (**A**) UV irradiated (20 J/m^2^) cells were allowed to recover for 6 hour. Representative images of UV treated cells display highly discontinuous, fragmented tails. The modified BrdU comet assay allowed proper differentiation between comet head and tail, which is essential for precise quantitation. Following the BrdU comet assay, the incorporated BrdU was identified by anti-BrdU primary antibody and cells were detected using Alexa Fluor 488 conjugated secondary antibody (green, panel I). Ethidium bromide counterstaining of the same cells (red, panel II) and merged images (yellow, panel III) are shown. (**B**) BrdU immunostaining detects S-phase cells without synchronisation. HCT116(RAD18^−/−^) cell were UV irradiated (40 J/m^2^) and allowed to recover for 6 hour. Staining was carried out as described in (A). Arrows indicate the non-S-phase cells which has not been stained with anti-BrdU antibody but only with ethidium bromide.

To improve the sensitivity of the alkaline BrdU comet assay we modified the lysis conditions. Instead of using double lysis we used a single step alkaline lysis with two detergents, 1% Triton X-100 and 0.5% sodium lauroyl sarcosinate. This modification ensured better resolution of the tail with farther migrated fragments and more extended loops, which thus became more distinguishable from the head, therefore the comet images obtained are suitable not only for software analysis but even for visual scoring (Figure 3). To avoid possible oxidative DNA damage, which can be caused by uncontrolled formation of free radicals during overnight incubation in aqueous solution at 37°C we applied a short, 90 minute single step lysis at 4°C. This improvement helps minimize possible artefacts that can be generated not only by free radicals, but also by the conversion of heat-labile sites to strand breaks that can happen during warm lysis [44]–[46]. We also introduced a washing step with PBS after lysis to ensure the removal of cell debris which, if not removed properly, results in protein precipitates during alkaline unwinding at pH 13, causing high background during immunostaining.

To directly compare the sensitivity of the alkaline BrdU comet PRR method to the basic comet assay in the detection of low molecular weight DNA we also visualised comets by ethidium bromide after immunostaining using anti-BrdU antibody. We found that the BrdU detection of the comets was much more sensitive as compared to the traditional ethidium bromide staining (Figure 3). Due to the weaker affinity of ethidium bromide to single-stranded DNA and the high degree of specificity of the anti-BrdU antibody, the BrdU immunolabelling was able to visualise even those shorter single-stranded DNA fragments, which were migrated much farther from the head and could not be visualised by ethidium bromide staining. Therefore, the BrdU comet method provides a better visualisation even for longer, more extensively spread comet tails, thereby providing a better sensitivity than the standard comet assay (Figure 3A and B).

### Measurement of Repair Kinetics in Cells Damaged in S-phase and in Non S-phase

Detectable level of BrdU pulse labelling of DNA occurs only in cells being in S-phase at the time of labelling, therefore, the cells being in S-phase and non S-phase can be distinguished, based on lack of BrdU immunostaining in non S-phase cells (Figure 3 B). Thus S-phase- and non S-phase cell specific DNA fragmentation can be measured simultaneously in the same cell population, which makes the method more user friendly since it eliminated the need for syncronisation of the cell culture.

In addition to following PRR of the S-phase cell population, the recovery from DNA damage in the non-S-phase pools of the cell population as visualized by the assembly of fragments into longer DNA molecule can also be followed at the very same time in ethidium bromide stained, BrdU-negative cells. Therefore, being a “two in one” method, the BrdU comet assay provides information on the effect of a given DNA damaging agent on the S-phase as well as the non-S-phase cell populations and can recognise those agents that specifically affect either of these cell populations as well as distinguish between DNA repair and PRR impairments.

### Analysis of the Dose-dependent Inhibition of Replication Fork Movement in RAD18 Knockout Human Cells by the BrdU Comet PRR Assay

**Figure 4:**
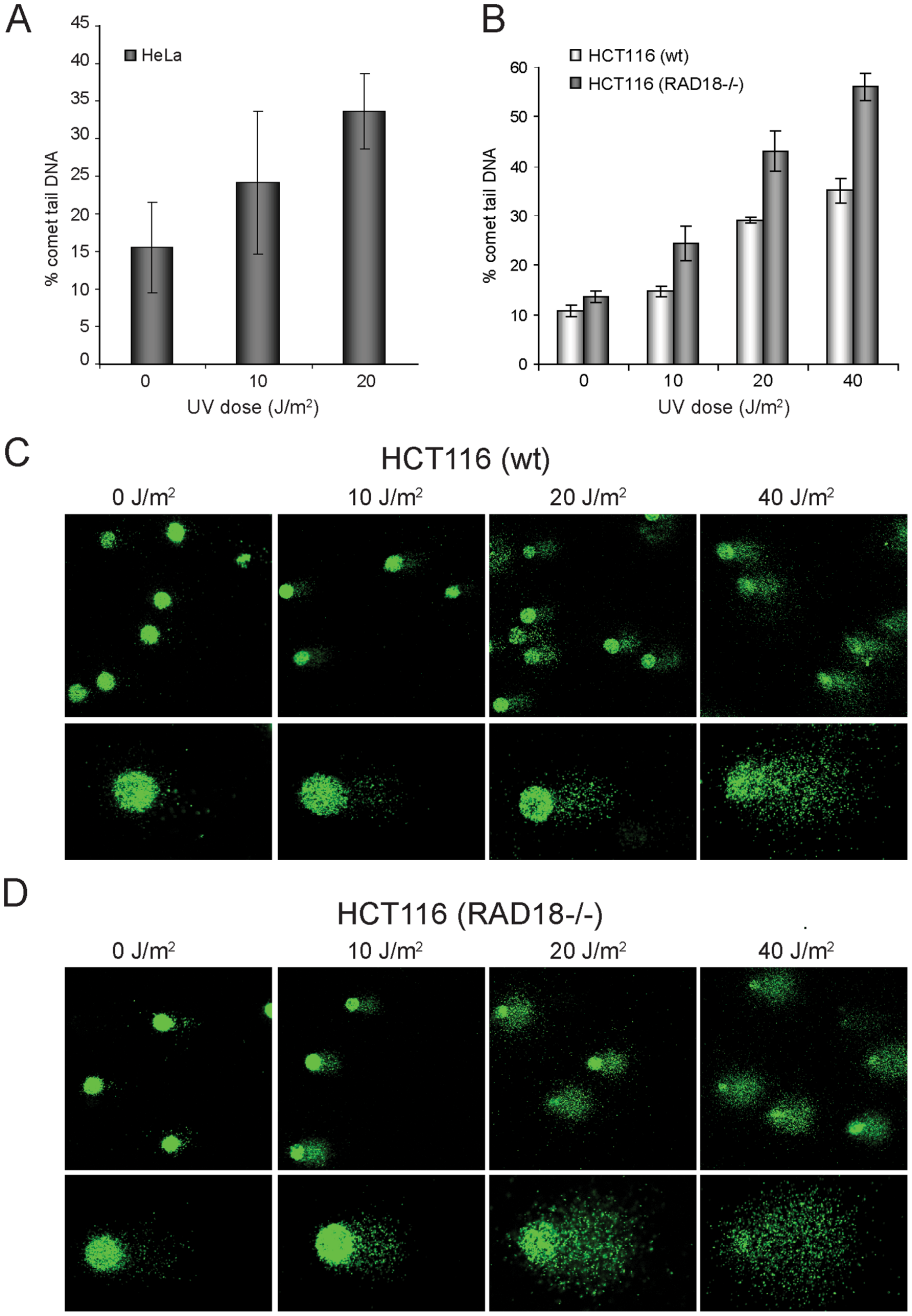
Dose dependent inhibition of replication progression caused by UV as detected by BrdU comet PRR assay. Cells were BrdU pulse labelled and mock treated or irradiated with UV and replication fork progression was followed by alkaline BrdU comet PRR assay. BrdU was detected by immunostaining, and quantitative measurement of comet tail DNA was done by Komet5 software. The % comet tail DNA, as a measure of discontinuity was illustrated. (**A**) Dose-effect curve of HeLa cells after 4 h recovery time. (**B**) Dose-effect curve of HCT116(wt), HCT116(Rad18^−/−^)cell lines after 6 hour recovery. Each data point represents the mean of three independent experiments. Error bars indicate standard deviations. (**C**) Representative alkaline BrdU comet PRR images as revealed by anti-BrdU immunostaining of HCT116(wt) cells irradiated with increased UV dose as indicated followed by 6 hour recovery. (**D**) As (C) but HCT116(Rad18^−/−^) cells were used instead of wild type cells.

Previous studies suggest an important role of RAD18 in post-replication repair [5], [12], [13], [15]. To gain more insight into the role of human RAD18 at a single cell level we tested replication fork progression with time upon UV damage in isogenic HCT116 wild-type and HCT116(RAD18^−/−^) cells using the alkaline BrdU comet PRR method. First, to determine the sensitivity of the method, we compared the inhibitory effect of different doses of UV on DNA elongation (Figure 4A and B). As compared to the HCT116 wild type cells in the HCT116(RAD18^−/−^) cell line we observed dose-dependent inhibition of replication fork elongation in both cell lines, but there remained significantly higher DNA fragmentation in HCT116(RAD18^−/−^) cells than in wild type cells at all tested UV doses after 6 h (Figure 4B).

**Figure 5:**
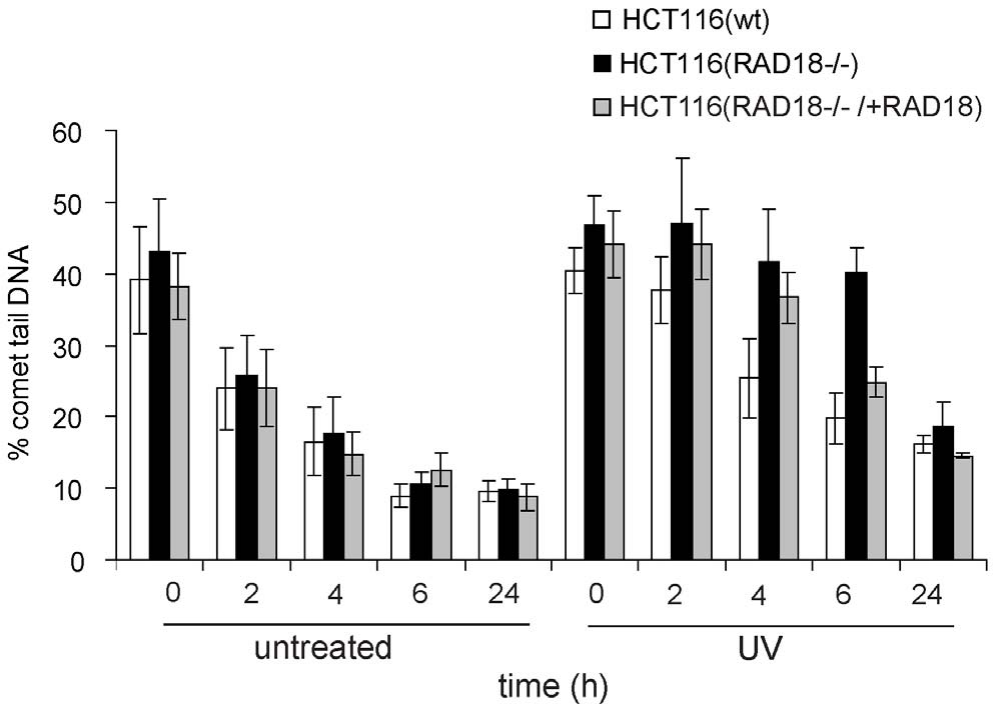
Time course curves of UV-irradiated HCT116(wt), HCT116(Rad18^−/−^), and HCT116(RAD18^−/−/^+RAD18), which is a HCT116(Rad18^−/−^) cell line expressing RAD18 for complementation. Cells were BrdU pulse labelled and mock treated or irradiated with UV (20 J/m^2^) and replication fork progression was followed by alkaline BrdU comet PRR assay. The % comet tail DNA, as a measure of discontinuity was monitored in untreated and in UV treated cells. Each data point represents the mean of three independent experiments. Error bars indicate standard deviations.

As shown by representative BrdU comet images in HCT116(RAD18^−/−^) cells at doses of 20 and 40 J/m^2^, the vast majority of cellular DNA migrated into the comet tail, reflecting the unmatured DNA as a consequence of fork stalling, whereas in the wild-type cells a significant amount of DNA remained in the comet head, representing the higher amount of matured DNA (Figures 4C and D). In contrast to damaged cells, in undamaged cells we found no difference in DNA maturation in the absence or presence of RAD18 (Figure 5). In the wild-type HCT116 cells the percentage of comet tail DNA was decreased by 50% after 6 h recovery time, representing the continuous conversion of fragmented DNA into matured, high molecular weight DNA. In marked contrast, DNA maturation was strongly inhibited in HCT116(RAD18^−/−^) cells since the percentage of comet tail DNA was decreased by only 10% even after 6 h, suggesting the prolonged existence of gaps in newly replicated DNA. This effect is attributed to the absence of RAD18 protein, since the complementation of RAD18 restored almost wild-type level of DNA maturation after 6 h of UV-irradiation (Figure 5). Importantly, we found that at 24 h after UV-irradiation HCT116(RAD18^−/−^) cells were also capable of restoring DNA continuity to normal levels, which indicates that in the absence of RAD18 other, though less efficient, other players can still provide the completion of replication of damaged DNA. Together these experiments provided further insight into the role of RAD18 in PRR as well as evaluation of the alkaline BrdU comet PRR assay as a reliable tool to address new gene functions.

## Discussion

Post-replication repair serves as a protective mechanism in damaged cells that allows the conversion of damage-induced single-strand gaps to high molecular weight DNA and thereby facilitates the completion of DNA replication without removing the replication-blocking lesions from template DNA. Therefore, these molecular events are also referred to as DNA damage tolerance pathways [3], [17]. PRR is also a part of the DNA damage response machinery, which is intertwined with numerous other fundamental biological processes such as cell cycle regulation, chromatin remodelling, apoptosis and carcinogenesis [47]. Elucidation of the PRR mechanisms that play an important role in the S-phase and to complete gap filling even in the G2-phase of the cell cycle could help in better understanding mutagenesis and carcinogenesis. In spite of the active interest and the significant investigative effort little is known about PRR in human cells. Even the contribution of the highly conserved players of the three branches of the human RAD18-dependent PRR, namely the error–prone- and error-free translesion synthesis, and the error-free template switching, remains to be further elucidated just like the characterization of the PRR role of the recently identified new players involved in DNA damage response such as HLTF, SHPRH, ZRANB3, FAN1 and Spartan [48]–[52]. Without doubt, there is a need for novel experimental approaches, which can unravel the unknown aspects of gene functions to PRR.

The currently known classical methods that measure PRR such as alkaline sucrose gradient sedimentation [53]–[55], alkaline DNA unwinding [56] and alkaline elution [57], [58] all involve radioactive labelling followed by alkaline ultracentrifugation to detect fragmented DNA based on size fractionation of DNA. Apart from radioactive labelling and long centrifugation procedures, these methods also have limitations, like requirement of large sample volumes, usage of whole cell lysates, laborious sample processing, insufficient sensitivity and none of them is suitable for single cell analysis. In contrast, the comet assay requires small amounts of cells and has high sensitivity in detecting even low levels of DNA fragmentation (50 strand breaks/diploid mammalian cell [59]). Furthermore, a BrdU labelling strategy prior to DNA damage treatment and a “one step” lysis at low temperature minimize artefact formation and makes the BrdU comet PRR assay described here a simple and very useful technique in assessing PRR.

PRR is centrally controlled by the RAD18 ubiquitin ligase, and its function have been implicated in PCNA monoubiquitylation-dependent pathways such as template switching and translesion synthesis [5], [12], [13], [15]. However, the PRR function of RAD18 has not been characterized at a single cell level. Therefore, RAD18 seemed a good candidate to test the reliability of the alkaline BrdU comet PRR method as a suitable PRR assay. Indeed, we confirmed by the alkaline BrdU comet PRR method that RAD18 knock out cells showed significant delay in replication fork progression on UV-damaged DNA as compared to the isogenic wild-type cells. Furthermore, we proved that the replication delay was demonstrably caused by the lack of RAD18, because RAD18 could complement the PRR impairment of HCT116(RAD18^−/−^) cells. On the other hand, in HCT116(RAD18^−/−^) cells without UV irradiation no replication delay was observed, and the kinetics of DNA replication and the maturation of nascent DNA were very similar to wild-type cells. These data reflect the specific requirement of RAD18 for an efficient PRR. In addition, our findings suggest the existence of possible backup mechanisms, which can operate in the absence of RAD18, since HCT116(RAD18^−/−^) cells were able to restore normal DNA elongation by 24 hours after DNA damage. Using the BrdU Comet PRR assay it will be interesting to explore this backup pathway by testing double knock down cells, in which RAD18 and other potential PRR gene knockdowns are combined.

Taken together, our results on the characterization of human RAD18 at a single cell level suggest that the BrdU comet PRR assay is highly applicable for investigators, who study replication of damaged DNA and PRR process.
